# Phylogeographic clustering of *Salmonella enterica* serovar Mississippi in the southeastern United States indicates regional transmission pathways

**DOI:** 10.1128/aem.02136-25

**Published:** 2026-01-27

**Authors:** Mel H. Yoshimoto, Lauren K. Hudson, Harleen K. Chaggar, Katie N. Garman, John R. Dunn, Agricola Odoi, Thomas G. Denes

**Affiliations:** 1 Department of Public Health, The University of Tennessee 161499, Knoxville, Tennessee, USA; 2 Department of Food Science, The University of Tennessee 736698, Knoxville, Tennessee, USA; 3 Tennessee Department of Health 5719, Nashville, Tennessee, USA; 4 Department of Biomedical and Diagnostic Sciences, The University of Tennessee 204922, Knoxville, Tennessee, USA; Centers for Disease Control and Prevention, Atlanta, Georgia, USA

**Keywords:** *Salmonella *Mississippi, *Salmonella*, phylogeography

## Abstract

*Salmonella enterica* subspecies *enterica* serovar Mississippi (*S*. Mississippi) is a polyphyletic serovar endemic in Australia, New Zealand, the United Kingdom, and the United States. From 2018 to 2024, it was the 13th most frequently reported clinical *Salmonella* serovar in the United States. Its incidence in the United States is geographically focused within the Southeast, with 78% of US cases in that region. The objective of this study was to determine the phylogeographical patterns of clinical *S*. Mississippi in the southeastern United States using sequenced clinical isolate data and metadata from 10 state public health laboratories in the region. Phylogenetic analysis, based on core single-nucleotide polymorphism (SNP) differences, resulted in five major clades (Ai, Aii, Bi, Bii, and C), four of which were consistent with previous studies. Clade Ai, comprising 99% of study isolates, was systematically divided into seven subclades. County-level mapping of clade Ai revealed distinct geographical distributions at the clade and subclade levels. For example, subclade Ai1 was predominantly distributed along the East Coast, while subclade Ai3 was primarily in western Tennessee. Moran’s *I* analysis revealed significant county-level spatial autocorrelation for clade Ai and its subclades, providing statistical evidence supporting the observed geographical clustering. Additionally, simple linear regression showed statistically significant (*P* < 0.01) positive associations between isolate-to-isolate genomic distance (core SNP differences) and county-to-county geographical distance (km) at the clade and subclade levels. Taken together, these findings provide additional layers of insight into the phylogeographical patterns of *S*. Mississippi.

**IMPORTANCE:**

*Salmonella* Mississippi is a significant public health concern in the southeastern United States; understanding its transmission dynamics is critical for improving surveillance and control. This study leverages a unique data set obtained through regional state public health laboratories, enabling county-level geographical analysis that provides finer resolution than previous studies. A novel, multifaceted approach was applied to characterize the phylogeography of *S*. Mississippi, integrating phylogenetic, spatial, and regression analyses. Moran’s *I* confirmed strong spatial autocorrelation, while regression analyses showed statistically significant positive associations between genomic and geographical distances. Collectively, these analyses revealed localized clustering, suggesting regional transmission pathways or enzootic reservoirs. Identifying sources or contributing factors could facilitate development and implementation of locally targeted control strategies. These findings provide insight into the spatial ecology of this serovar and establish a framework for future primary-base studies to develop models based on more predictors and conduct more detailed investigations of ecological and epidemiological predictors.

## INTRODUCTION

*Salmonella enterica* subspecies *enterica* serovar Mississippi (*S*. Mississippi) was the 13th most frequently reported clinical *Salmonella* serovar in the United States according to Report Data from 2018 to 2024 obtained from the BEAM Dashboard (1). *S*. Mississippi is a nontyphoidal serovar but has been found to encode the *Salmonella* cytolethal distending toxin (S-CDT, also called “typhoid toxin”). S-CDT is a genotoxin that may cause invasive systemic disease, persistent asymptomatic bacteremia, DNA damage, and other forms of long-term sequelae (2, 3). Rare but recorded sequelae caused by *S*. Mississippi include renal abscess in an immunocompetent patient (4) and transplacental transmission resulting in a spontaneous miscarriage at 18 weeks of gestation (5).

Existing literature suggests that *S*. Mississippi is a polyphyletic serovar endemic in Australia (AUS), New Zealand, the United Kingdom, and the United States (6–8). In Tasmania (AUS), the serovar has demonstrated wide genetic diversity and persistence in the environment, with a broad range of host reservoirs that persist over time (8–10). Previous phylogenetic analysis by Cheng et al. compared whole-genome sequence (WGS) data of *S*. Mississippi isolates with other *Salmonella enterica* serovars. They identified two primary clades: clade A, within section Typhi in *S. enterica* clade A, and clade B, within *S. enterica* clade B. Their results suggest that clades of *S*. Mississippi evolved from different ancestors and that isolates cluster geographically. Clade A isolates were primarily from the United States (subclade Ai) and Australia (subclade Aii). Clade B isolates were primarily from the United Kingdom (subclades Bi and Bii) (6). The number of polyphyletic serovars known to us continues to increase as new *Salmonella* isolates continue to be sequenced (6).

*S*. Mississippi has been a frequently reported serovar since 2010. Historically, this serovar has been geographically focused within the southeastern United States (11) and the Gulf Coast states (12). Incidence of *S*. Mississippi is highest along the Mississippi River and the Mississippi-Louisiana coast (11, 13). From 2018 to 2024, 77.7% of all *S*. Mississippi cases in the United States occurred in the Southeast (Alabama [AL], Arkansas [AR], Florida [FL], Georgia [GA], Kentucky [KY], Louisiana [LA], Mississippi [MS], North Carolina [NC], South Carolina [SC], Tennessee [TN], and Virginia [VA]) (1). The highest incidence rates of culture-confirmed human *S*. Mississippi infections (per 100,000 population) in 2016 were in MS (4.2), LA (2.1), and NC (1.0), followed by AL (0.8), TN (0.6), AR (0.5), and GA (0.3) (11). KY (0.1), SC (0.1), VA (0.1), and FL (0.02) had lower incidence relative to other southeastern states (11). Geographical restriction or distribution may indicate association with local habits or food products, persistence in or adaptation to particular natural environments, and/or animal reservoirs within a specific habitat (12, 14–16).

*S*. Mississippi may have higher transmission rates from animal or environmental sources than food (8, 17, 18). According to National Outbreak Reporting System data obtained from the BEAM Dashboard, there have been only four outbreaks caused by *S*. Mississippi with confirmed etiology (and two with suspected etiology) (19). Of those, only one lists a food vehicle: a 2022 multistate outbreak caused by tomatoes resulting in 102 illnesses and 23 hospitalizations. Among the top 20 nontyphoidal *Salmonella* serovars in the United States, *S*. Mississippi had the lowest foodborne relatedness measure (0.01) (20). This measure indicates a low association with foodborne transmission. Additionally, *S*. Mississippi is not commonly isolated from USDA-FSIS-regulated products, further supporting that this serovar does not likely have a food-animal reservoir (21–24).

Previous *S*. Mississippi studies show evidence of human infection from wildlife. This serovar has been associated with or isolated from zoonotic reservoirs, including wild animal (7, 13), avian (8, 13), amphibian, reptile (13), and equine (6, 13) sources. Other environmental sources identified include untreated drinking water (7, 8) and untreated recreational water (2018 outbreak in Alabama associated with a lake/reservoir) (19). Untreated freshwater reservoirs and watersheds, such as the Mississippi River, may serve as natural reservoirs for *S*. Mississippi (7, 8, 25).

Additionally, *S*. Mississippi exhibits high seasonality, with summer peaks exceeding other *S. enterica* serovars (7, 8, 12). This temporal increase could be related to increased presence of animal hosts (e.g., reptiles and amphibians), increased exposure to a source (e.g., recreational water), or increased pathogen levels in a continually present reservoir due to increasing favorable environmental conditions (12, 26). Identifying control strategies for this serovar is difficult due to its high genetic diversity and wide range of animal and environmental sources (7, 8, 13, 14).

The purpose of this study was to better understand spatial patterns of reported and sequenced *S*. Mississippi clinical isolates in the southeastern United States and describe the relationship between genomic distance and geographical distance. This study leverages a unique data set of *S*. Mississippi isolates sequenced at 10 state public health laboratories (SPHLs) in the Southeast, obtained through established data use agreements with those SPHLs, enabling county-level geographical analysis that provides finer resolution than previous studies conducted at the state or national level (6, 7). A novel, multifaceted approach was used to assess the county-level phylogeography of *S*. Mississippi in the southeastern USA: (i) phylogeny was determined; (ii) county-level mapping was performed to provide increased resolution on how this serovar is distributed within the region; (iii) spatial autocorrelation was assessed using Moran’s *I* to statistically evaluate the clustering patterns within the region; and (iv) simple linear regression (SLR) was used to determine if there were associations between genomic and geographical distances (and the directions of the associations, if any).

## RESULTS

### Phylogeny consists of five clades

**Fig 1:**
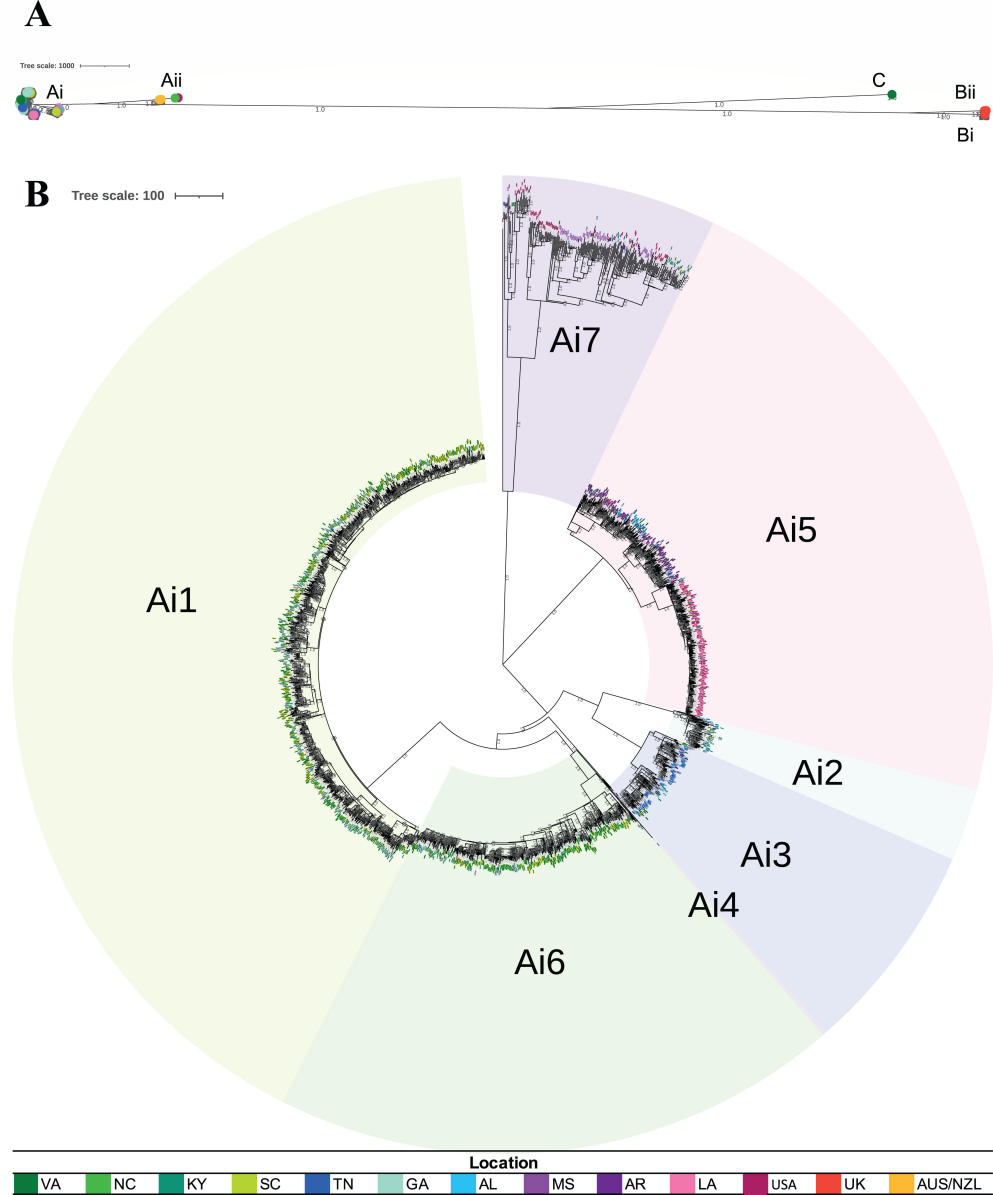
Neighbor-joining phylogenetic trees of all isolates and of clade Ai with subclades. Both trees were constructed in MEGAX (27, 28) using the neighbor-joining method (29) and visualized and annotated using iTOL (30). The trees are drawn to scale, with branch lengths representing the number of base differences at core single-nucleotide polymorphism (SNP) positions. The evolutionary distances were computed using the number of differences method (31). All ambiguous positions were removed for each sequence pair (pairwise deletion option). Isolate locations are indicated by colored circles or text at tree tips (see legend at the bottom). (**A**) Phylogeny of all isolates. This analysis involved 2,853 isolates and a total of 55,550 core SNP positions. The proportion of replicate trees in which the associated taxa clustered together in the bootstrap test (50 replicates) is shown next to the branches if ≥0.75 (32). (**B**) Phylogeny of clade Ai. This analysis involved 2,775 isolates and a total of 40,891 core SNP positions. The proportion of replicate trees in which the associated taxa clustered together in the bootstrap test (100 replicates) is shown next to the branches if ≥0.75 (32). Subclades are indicated.

Phylogenetic analyses of the study isolates (*n* = 2,797) and reference genomes (*n* = 56) revealed five primary clades: Ai, Aii, Bi, Bii, and C (Fig. 1A; Table 1). This shows that *S*. Mississippi is a polyphyletic serovar, consistent with the results of Cheng et al. (6). Four of the identified clades (Ai, Aii, Bi, and Bii) correspond to those identified by Cheng et al. However, an additional fifth clade (C) was identified that was not observed by Cheng and colleagues. Notably, clade C only contained three isolates. Clades Ai and Aii are more closely related, with clade C falling between A clades and B clades. Clade Aii contained only one study isolate (from NC), and the reference genomes in this clade (*n* = 19) were primarily from New Zealand (*n* = 9) and Australia (*n* = 9). Clade Bi contained 22 study isolates (from all included states, except AL and AR). The reference genomes in this clade (*n* = 16) were primarily from the United States (*n* = 15). Clade Bii contained no study isolates, and all the reference genomes contained in this clade (*n* = 7) were from the United Kingdom. Clade C contained only three study isolates, all from VA (6).

**TABLE 1:** Count of sequenced *S*. Mississippi clinical isolates per clade and subclade

Clade/subclade	No. of study isolates	No. of reference isolates	Total no. of isolates
Ai	2,761	14	2,775
Ai1	1,155	5	1,160
Ai2	67	0	67
Ai3	197	0	197
Ai4	4	0	4
Ai5	619	2	621
Ai6	522	5	527
Ai7	197	2	199
Aii	1	19	20
Bi	22	16	38
Bii	0	7	7
C	3	0	3
Total	2,787	56	2,843

Clade Ai contained approximately 99% of the study isolates (*n* = 2,761) and reference genomes from the United States (*n* = 14). This is consistent with the results of Cheng et al. (6), who found that clade Ai isolates were predominantly from the United States. Further analyses focused on this clade. Clade Ai was systematically divided, resulting in seven Ai subclades (Fig. 1B; Table 1), with the majority of isolates in subclade Ai1 (*n* = 1,160). Subclade Ai7 was the most genetically diverse (within subclade average distance: 310 single-nucleotide polymorphisms [SNPs]; range: 0–1,192 SNPs), followed by Ai5 (average: 119 SNPs, range: 0–209 SNPs), Ai3 (average: 98 SNPs, range: 0–165 SNPs), Ai1 (average: 96 SNPs, range: 0–156 SNPs), Ai6 (average: 93 SNPs, range: 0–441 SNPs), Ai2 (average: 72 SNPs, range: 0–148 SNPs), and Ai4 (average: 53 SNPs, range: 45–59 SNPs).

Subclades Ai1 and Ai6 isolates were primarily from VA, NC, SC, and GA. Subclade Ai2 isolates were primarily from GA, with a small but notable portion of isolates from TN and AL. Subclade Ai3 isolates were primarily from TN, with several isolates from VA and KY. Subclade Ai4 isolates were from TN, MS, and GA. Close to half of subclade Ai5 isolates were from LA, with the other half predominantly from MS followed by AL and TN. Subclade Ai7 isolates were primarily from AR and LA, followed by GA.

### County-level geospatial patterns

**Fig 2:**
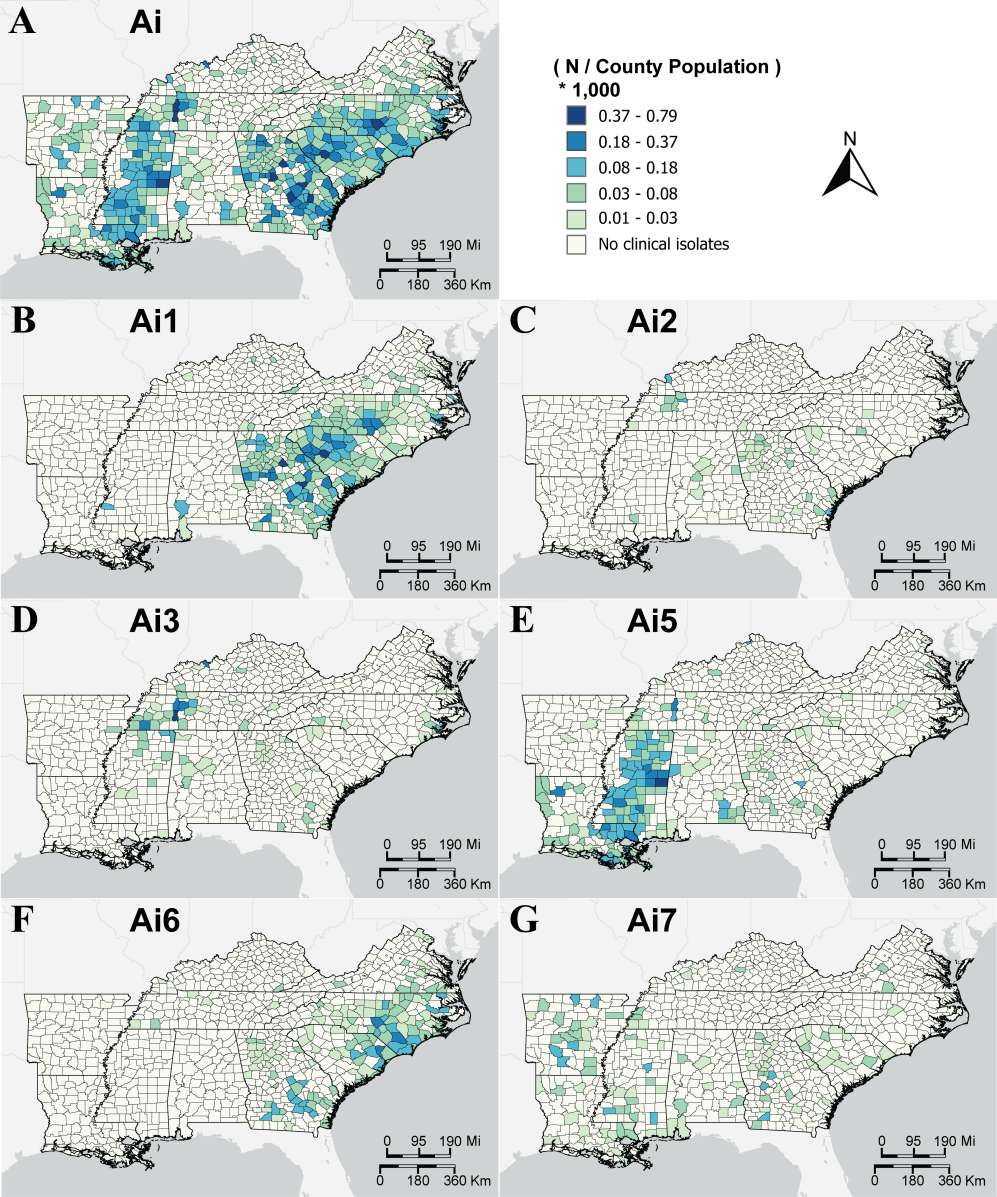
County-level incidence risk of *S*. Mississippi clinical isolates by clade. Counties are shaded by number of reported and sequenced *S*. Mississippi isolates per county population (see the legend at top right) for (**A**) all of clade Ai and (**B–G**) subclades Ai1–Ai7, excluding subclade Ai4. This includes all relevant study isolates, which had isolation years from 2000 to 2022.

County-level incidence risk (IR) of sequenced *S*. Mississippi clinical isolates was mapped at the clade and subclade levels (Fig. 2A through G). Dohoo et al. define incidence risk as “the probability that an individual will contract or develop a disease in a defined time period” (33). In the current study, IR is the number of sequenced clinical *S*. Mississippi isolates from each county divided by the county population, scaled by a multiplier of 1,000. The population area includes the following southeastern states: AL, AR, GA, KY, LA, MS, NC, SC, TN, and VA. Isolates were collected from 2002 to 2022, the majority collected between 2017 and 2022. It is important to note that the IR reported in the current study represents observed or apparent IR rather than true IR. This measure reflects the number of isolates received and sequenced by SPHLs. Additionally, variability in healthcare access, diagnostic practices, reporting, and health infrastructure across jurisdictions may influence the number of isolates reported from different areas. For example, underreporting may occur in areas with limited access to healthcare, potentially leading to lower observed IR despite actual disease occurrence. As such, higher or lower observed IR in certain counties may reflect differences in reporting rather than actual differences in disease occurrence.

Clade Ai clustered in two geographical regions of the southeastern United States (Fig. 2A). One higher IR region runs from west TN to southeast LA, covering northeast to southwest MS and following the Mississippi River (on the MS side) except for a portion of northwest MS. The other higher IR region runs throughout states bordering the East Coast, from the VA-NC border, throughout most counties in SC, and along the SC-GA border. The highest IR was observed in south-central NC, central SC, and southeastern GA.

Close examination of the geographical distribution of subclades of clade Ai reveals that subclades Ai1, Ai3, Ai5, and Ai6 had the strongest evidence of spatial clustering in the southeastern United States (Fig. 2B through G). There is overlap in the geographical distribution of subclades Ai1 and Ai6. Subclade Ai1 (Fig. 2B) was predominantly distributed along the East Coast from southern VA through SC and most of GA, with other occurrences in Clarke and Baldwin counties in AL and Jefferson County in MS. Subclade Ai6 (Fig. 2F) exhibited a strong presence along the Atlantic Coastal Plain from southeast VA to southeast GA, with inland distribution in NC, SC, and GA, particularly notable in NC. This subclade also had a sparse and scattered distribution throughout TN, KY, and inland VA.

There is also overlap in the geographical distribution of subclades Ai3 and Ai5. Subclade Ai3 (Fig. 2D) was primarily clustered in western TN and Alcorn and Tishomingo counties in MS, Hancock County in KY, and Pamlico County in NC. Subclade Ai5 (Fig. 2E) was observed in west-central TN through northeast MS to southeast LA (mostly in the southern deltas, but also the Mississippi alluvial plain), with a moderate presence in northwest LA counties and southeastern AL.

In comparison, the distributions of subclades Ai2 and Ai7, in addition to being less common, were more sporadic spatially. Subclade Ai2 (Fig. 2C) was sparsely distributed throughout KY, TN, NC, MS, AL, and GA, with the most notable presence in northern GA and west-central TN. The highest IR was observed in Livingston County in KY (the only county in KY for this subclade), Houston County in TN, and McIntosh County in GA. The distribution of subclade Ai7 (Fig. 2G) was random throughout the region, similar to subclades Ai2 and Ai4, with exceptions in southwest MS and central AR. The counties with the highest IR in Ai7 were in AR, MS, and GA. In AR and GA, the highest observed IR was in densely populated counties, whereas in MS, the highest observed IR was in rural counties. These findings highlight that higher IR is not consistently associated with population density and may vary, depending on other factors.

Subclade Ai4 (Fig. S1A) contained only four total isolates and was limited to Tate and Yalobusha counties in MS and Fayette County in TN.

### Spatial clustering

Using queen contiguity weights, there was significant global spatial autocorrelation of sequenced clinical *S*. Mississippi isolates at the county level. The Global Moran test of spatial autocorrelation (Moran’s *I*) was statistically significant (*P* < 0.05) at the clade and subclade levels for both the empirical Bayes (EB) adjusted and univariate Moran’s *I* (Table 2). The degree of clustering measured using Moran’s *I* is consistent with the visual inspection of county-level geospatial patterns (Fig. 2A through G; Fig. S1A). The empirical Bayes adjusted Moran’s *I* directly standardizes the county-level frequency of *S*. Mississippi isolates using the base variable of county-level population. The population multiplier used in the IR maps (Fig. 2A through G; Fig. S1A) is not applied to the calculation of global spatial autocorrelation (Table 2).

**TABLE 2:** Moran’s *I*^*a*^

Clade	Empirical Bayes adjusted		Univariate	
	Moran’s *I*	*P* value	Moran’s *I*	*P* value
Ai	0.382	0.01	0.279	0.01
Ai1	0.443	0.01	0.398	0.01
Ai2	0.187	0.01	0.273	0.01
Ai3	0.214	0.01	0.097	0.01
Ai4	−0.002	0.16	−0.003	0.21
Ai5	0.499	0.01	0.252	0.01
Ai6	0.491	0.01	0.381	0.01
Ai7	0.085	0.02	0.174	0.01

^*a*^ *P* value tested using 99 permutations.

Subclades Ai5 (*I* = 0.499) (Fig. 2E), Ai6 (*I* = 0.491) (Fig. 2F), Ai1 (*I* = 0.443) (Fig. 2B), clade Ai (*I* = 0.382) (Fig. 2A), and subclade Ai3 (*I* = 0.214) (Fig. 2D) had the largest magnitudes of spatial clustering (Table 2) with distinct spatial distributions (Fig. 2A through G). Putting Moran’s *I* values in the context of their respective maps, we found that the aforementioned subclades had concentrated spatial distributions. For example, the frequency of subclade Ai5 isolates (Fig. 2E) had a focused presence throughout MS and the largest magnitude of spatial clustering (*I* = 0.499). Clade Ai (Fig. 2A) had a slightly smaller magnitude of spatial clustering (*I* = 0.382) compared to subclade Ai5. Clade Ai isolates, which include all subclade isolates, were more spread out than subclade Ai5 and had two clear clusters. One spans from west TN down through MS; the other spans from the Carolinas (NC and SC) down through GA.

Subclades Ai2 (*I* = 0.187) (Fig. 2C), Ai7 (*I* = 0.085) (Fig. 2G), and Ai4 (*I* = −0.002) (Fig. S1A) had the smallest magnitudes of spatial clustering (Table 2). Upon visual inspection (Fig. 2A through G; Fig. S1A), these subclades appear to be more spread across the region compared to subclades with a larger Moran’s *I*. The empirical Bayes adjusted Moran’s *I* for subclades Ai2 and Ai7 is lower than the univariate Moran’s *I*. For all other subclades and clade Ai, the adjusted Moran’s *I* was slightly larger than the univariate value (Table 2). Subclade Ai4 is close to 0 (*I* = −0.002) and was not significant (*P* > 0.05), indicating that the isolates in Ai4 are randomly dispersed. This result can be attributed to the small number of isolates within subclade Ai4 (*n* = 6).

### Association between geographical and genomic distances

Varying degrees of weak positive correlation are observed between genomic distance (the number core SNP differences) and geographical distance (km) for clade Ai and its subclades (Table 3; Fig. S2). The phylogenetic structure of and diversity within each group of isolates influences the distribution of isolate-to-isolate SNP differences. For most of the subclades, there are distinct groupings along the *x*-axis (multimodal distribution) due to distinct subpopulations within the subclade (Fig. S2B through G). These groupings are also present in the clade Ai scatterplot but are more difficult to see due to the large number of observations (Fig. S2A). Additionally, the paired nature of geographical distance and genetic distance from isolate-to-isolate comparisons makes for a large data set where overplotting is inevitable.

**TABLE 3:** Box-Cox transformation and simple linear regression

Clade	Box-Cox	Simple linear regression								
	Transformation (*λ*)	*F*-test	*p*_model_	Bonferroni adjusted *p*_model_	Intercept (*β*_0_)	*p*_intercept_	Slope (β_1_)	*p*_slope_	Formula	Pearson’s *r*
Ai	sqrt^*a*^ (0.505)	*F*_(1, 319350)_ = 4,064	<2.20 × 10^−16^		14.5	<2.20 × 10^−16^	0.00863	<2.20 × 10^−16^	*y* = 14.5 + 0.00863*x*	0.112
Ai1	sqrt (0.586)	*F*_(1, 170794)_ = 621.6	<2.20 × 10^−16^	<1.54 × 10^−15^	14.4	<2.20 × 10^−16^	0.00906	<2.20 × 10^−16^	*y* = 14.4 + 0.00906*x*	0.0602
Ai2	sqrt (0.465)	*F*_(1, 1532)_ = 56.02	1.21 × 10^−13^	8.44 × 10^−13^	10.5	<2.20 × 10^−16^	0.0349	1.20 × 10^−13^	*y* = 10.5 + 0.0349*x*	0.188
Ai3	sqrt (0.384)	*F*_(1, 17952)_ = 446.5	<2.20 × 10^−16^	<1.54 × 10^−15^	11.4	<2.20 × 10^−16^	0.0246	<2.20 × 10^−16^	*y* = 11.4 + 0.0246*x*	0.156
Ai4	log^*b*^ (0.0202)	*F*_(1, 4)_ = 1.548	2.81 × 10^−1^	1.97 × 10^0^	0.894	3.33 × 10^−1^	0.0109	2.81 × 10^−1^	*y* = 0.894 + 0.0109*x*	0.528
Ai5	sqrt (0.424)	*F*_(1, 46248)_ = 3,412	<2.20 × 10^−16^	<1.54 × 10^−15^	11.2	<2.20 × 10^−16^	0.0284	<2.20 × 10^−16^	*y* = 11.2 + 0.0284*x*	0.262
Ai6	sqrt (0.424)	*F*_(1, 72660)_ = 59.71	1.11 × 10^−14^	7.78 × 10^−14^	15.5	<2.20 × 10^−16^	0.00304	1.11 × 10^−14^	*y* = 15.5 + 0.00304*x*	0.0287
Ai7	sqrt (0.343)	*F*_(1, 10148)_ = 93.12	<2.20 × 10^−16^	<1.54 × 10^−15^	15.1	<2.20 × 10^−16^	0.00412	<2.20 × 10^−16^	*y* = 15.1 + 0.00412*x*	0.0954

^*a*^ Square root, formula: √km.

^*b*^ Logarithmic, formula: log_10_km*.*

The association between the pairwise geographical distance in kilometers (response/dependent variable) and pairwise genomic distance in number of core SNP differences (predictor/independent variable) was measured using SLR for individual subclades within clade Ai and for clade Ai as a whole. The Pearson correlation coefficient, *r*, is reported at the clade and subclade level (Table 3). The distribution and variance of the initial model’s residuals were not normally distributed, requiring a power transformation of geographical distance. The Box-Cox transformation method identified the square root transformation for clade Ai and subclades Ai1, Ai2, Ai3, Ai5, Ai6, and Ai7 to improve the distribution of the residuals, increasing the validity of statistical inference.

Pearson’s *r* revealed significant positive correlations (Table 3) at the clade and subclade levels, except subclade Ai4. Simple linear regression of all subclades, except subclade Ai4 (Fig. S1B), showed statistically significant positive associations between genomic and geographical distances (Table 3). The regression coefficient represents the change in the square root of geographical distance in kilometers per one unit increase in SNP difference. The lack of significant association for subclade Ai4 might be attributed to the small sample size (six observations). There was evidence of a significant association between genomic and geographical distances even when all clade Ai subclades were combined. When comparing the clade Ai data containing only within-group (within each subclade) comparisons to the clade Ai data with between-group (between-subclades) comparisons, the data with between-group comparisons had a slightly larger positive magnitude. SLRs for both data sets had a small *P* value (*P* < 0.001). It is suspected that the genomic diversity of *S*. Mississippi and pairwise isolate data amplify the amount of noise at the clade and subclade levels, resulting in low Pearson’s *r* values. Additionally, low Pearson’s *r* values suggest that there are independent variables missing from the model.

## DISCUSSION

The objective of this study was to determine the phylogeographical patterns of reported and sequenced clinical *S.* Mississippi in the southeastern United States using a large data set of clinical isolates from this region (*n* = 2,739). The large data set is an important strength of this study; it was obtained through established data use agreements with 10 SPHLs and allowed for high-resolution spatial analysis. It was found that *S*. Mississippi shows distinct geographical associations at the clade and subclade levels. The geographical clustering of this serovar within the southeastern United States suggests local or regional transmission pathways and reservoirs, which could be related to environmental exposures or regional food exposures (26). This pattern differs from other serovars that show relatively uniform geographical distribution, such as Typhimurium or Enteritidis, indicating uniform sources that are widely dispersed (e.g., nationally distributed food products) or a balanced mix of multiple sources of exposure (12). Surveillance data showing that *S*. Mississippi appears to be more associated with sporadically acquired infections than outbreak associated (only 0.83% clinical isolates from 2018 to 2024) (1) further supports that exposures are more likely to be environmental (34). Some potential environmental or ecological factors contributing to the observed spatial distribution in this study are principal aquifers (35), watersheds (36), agriculture (37), bird migration patterns (7), amphibian breeding (14, 26), population densities (37), temperature (38, 39), or ecosystem categorization (e.g., wetland type) (40).

Results from the test for global spatial autocorrelation (Moran’s I) suggest that the clustering observed in clade Ai and subclades Ai1, Ai2, Ai3, Ai5, Ai6, and Ai7 is unlikely to have occurred by chance. Supporting the visual and statistical observations of county-level geographical clustering, significant positive associations between genomic and geographical distances were found at both the clade and subclade levels. Clustering observed in subclade Ai4, where *n* = 6, was not statistically significant. Based on these findings, there is evidence of significant positive associations between genomic and geographical distances. Since the objective of this study was to assess if there was an association between genomic and geographical distances and not to estimate the effect, future studies using more detailed primary data are warranted. Such primary-base studies will need to investigate other factors with a focus on prediction and effect estimation. Additionally, further research is needed to understand the mechanism(s) of this positive relationship and the variation in correlation across subclades.

As genomic-based methods are being developed and refined for public health implementation, there is a need to determine the optimal level of genomic resolution (e.g., genomic distance thresholds) for subtyping of specific pathogen species or serovars for surveillance applications (e.g., cluster detection) (41). This is also needed for studies looking at “functional clades” similar to the current study (at what level of relatedness can geospatial and other trends be seen?). Cheng et al. found that this serovar has associations with differing countries at the clade level: Ai with the United States, Aii with Australia, and Bi and Bii with the United Kingdom (6). The findings here extend the work by Cheng et al. (6) on the phylogeny of this serovar, with a specific focus on the population structure of isolates from the southeastern United States, which are predominantly part of clade Ai. The subclade-level subtyping employed in the current study shows that this finer level of resolution reveals functionally relevant subclades where distinct geographical clustering is evident, which could be used to inform regional- and state-level public health response. For example, the phylogeographical distributions identified in the current study could be used by public health professionals, along with epidemiological data, to determine the potential geographical origin of a contamination or exposure event based on what clade an isolate belongs to (6, 42, 43).

County-level spatial analysis of clinical isolates and measuring the association between genomic and geographical distances for this serovar will allow for future exploration and identification of localized environmental risks. Environmental sampling to isolate and characterize environmental *S*. Mississippi isolates, similar to the nationwide genomic atlas of soil-dwelling *Listeria* developed by Liao et al. (44), could assist in identifying risk factors and natural reservoirs. Additionally, as temporal differences were not taken into account in the current study, it would be helpful to assess temporal incidence and climate factors (e.g., rainfall and temperature) or look at environmental factors that have a spatial pattern (e.g., land cover indices for water) to determine any correlations with incidence of human disease.

Identifying control strategies for polyphyletic serovars like *S*. Mississippi can prove difficult, as they are typically associated with a wide range of animal and environmental sources (7, 8, 13, 14). For other polyphyletic serovars, research has shown that different lineages can be associated with different hosts (6, 45, 46). A better understanding of sources of exposure will facilitate the development and implementation of targeted education and control strategies and interventions that limit further exposure and prevent illness. Additionally, knowledge of transmission pathways can aid in the design of questionnaires to gather relevant risk factor and exposure data and earlier identification of outbreak sources, leading to a more robust public health response.

The transition to WGS for surveillance of enteric pathogens has created an opportunity to integrate epidemiological and high-resolution genomic data to better understand pathogen transmission dynamics, as was done in the current study and can be applied to other *Salmonella* serovars and foodborne pathogens. This approach can also be used to elucidate sources or reservoirs with a more targeted subgroup of pathogens, such as reoccurring, emerging, or persisting (REP) strains, a new classification used by the CDC to track strains that cause illnesses over longer periods of time than an acute outbreak (47).

One limitation of this study is the use of isolates obtained through case-based passive surveillance, which is susceptible to underreporting or other biases. Also, because isolate data were obtained from different state public health departments, there may be differences between their surveillance, isolate submission, characterization, data collection, or reporting practices (e.g., for county of isolation, some states may use the county where the isolate was collected, and others may use the county of residence of the case). There are also likely differences between the locations studied (e.g., socioeconomic status, access to medical care, quality of medical care, and diagnostic methods) that could affect the representativeness of the data. Additionally, exact exposure information was not available, so, in some cases, county of isolation may not be where the exposure occurred.

### Conclusions

In this study, a novel, multifaceted approach was used to examine the county-level phylogeography of reported and sequenced *S*. Mississippi in the southeastern United States, a region where this serovar is geographically focused. This included characterizing its distribution and clustering patterns within the region and performing regression-based analyses to assess associations between genomic and geographical distances. Distinct phylogeographical distributions were observed for this serovar at both the clade and subclade levels. This clustering was further supported by the detection of significant (*P* < 0.05) county-level spatial autocorrelation, indicating that this serovar was not randomly distributed across the region. Significant (*P* < 0.01) positive associations between genomic distance (isolate-to-isolate core SNP differences) and geographical distance (square root of county-to-county distance [km]) were identified at both the clade and subclade levels. Collectively, these findings reveal localized clustering of *S*. Mississippi within the southeastern United States, which suggests local or regional transmission pathways and reservoirs that could be related to environmental or regional food exposures. The results of this study provide insights into the phylogeography of this serovar and provide a basis to further investigate and identify possible environmental sources, enzootic reservoirs, climate factors, or other risk factors contributing to human infection with this serovar within the southeastern United States. Further work in this area should involve model development, including exploring more potential predictors, to strengthen both prediction and effect estimation. These future efforts can, in turn, support the development and implementation of evidence-based, locally targeted control strategies. Further development of a model assessing associations between genomic and geographical distances could be leveraged by public health professionals to use genomic distances to predict geographical locations of exposures resulting in illness. Principal component analysis with the addition of environmental factors would be beneficial, given the large dimensions of data, resulting from the pairwise isolate-to-isolate comparisons of genomic and geographical distances.

## MATERIALS AND METHODS

### Isolate information

A multistate exploration of sequenced *Salmonella* Mississippi clinical isolates was conducted by collaborating with SPHLs in the southeastern United States (AL, AR, GA, KY, LA, MS, NC, SC, TN, and VA). The following information was requested from each SPHL on all sequenced *S*. Mississippi clinical isolates received up to 2022: isolate identifiers (PNUSA, SRR, or SAMN), source county, and year of isolation (*n* = 2,903) (Table 4; Table S1, Fig. S3). For AL, LA, and VA, the source county is the county of isolate collection. For AR, GA, KY, MS, NC, SC, and TN, the source county is the county of residence. The isolation years spanned from 2000 to 2022. NCBI E-utilities were used to retrieve SRR identifiers for isolates where only PNUSA was provided (*n* = 2,845). Fasterq-dump (v.2.11.2, part of SRA Toolkit) (48) was used to retrieve raw reads FASTA files (*n* = 2,801) from the NCBI SRA database. The raw reads were trimmed using Trimmomatic (v.0.39; Phred: 33, leading: 3, trailing: 3, sliding window: 4:15, and min len: 36) (49). Quality control was performed using FastQC (v.0.11.9) (50) and MultiQC (v.1.11) (51) Trimmed reads were assembled into contigs using SPAdes (v.3.13.1 [52, 53], with the careful option). Contigs with a length of <1 kb or coverage of <5× were removed from assemblies (54). QUAST (v.5.0.2 [55]) was used to generate assembly statistics.

**TABLE 4:** Count of sequenced *S*. Mississippi clinical isolates per state, including no. of isolates with known source county

State	No. of isolates			
	Total	Raw reads available	County known	Raw reads available and county known
Alabama	61	57	61	57
Arkansas	75	58	73	58
Georgia	688	673	648	634
Kentucky	15	15	15	15
Louisiana	388	388	387	387
Mississippi	229	229	229	229
North Carolina	627	605	611	593
South Carolina	465	465	459	459
Tennessee	194	191	194	191
Virginia	109	66	85	66
Other/unknown	52	50	0	0
Total	2,903	2,797	2,762	2,689

### Phylogenetic analyses

A total of 56 reference genomes were included in the phylogenetic analysis (Table 1; Table S2). Nineteen RefSeq assemblies of *S*. Mississippi isolates were downloaded from NCBI. Thirty-seven reference genomes from Cheng et al. (6) were selected, downloaded from NCBI, and included in the analysis to place the current study isolates in the context of already established clades.

Reference-free SNP detection was performed using KSNP (v.3.1, *k* = 19 [56–58]) with the assembled isolates (*n* = 2,801) and reference genomes retrieved from NCBI (*n* = 56). The resulting core SNP matrix fasta file was processed in MEGA (v.10.2.6 [27]) to construct a neighbor-joining phylogenetic tree with 50 bootstrap replicates. The tree was further visualized and annotated in iTOL (30, 59). Seven of the isolates did not fall into any of the clades previously established by Cheng et al. (6), so each was further evaluated by checking its computed serotype and antigen formula on NCBI Pathogen Detection (60), the computed serotype of closely related isolates in its SNP cluster on NCBI Pathogen Detection (60), and its SISTR1 (61) and SeqSero2 (62) predicted serotypes on EnteroBase (63). Three were predicted to be serovar Mississippi, and those were later assigned to a novel clade designated as clade C. The other four were predicted to be other serovars (Javiana, Enteritidis, Muenchen, and Saintpaul) and were removed from further analyses.

Reference-free SNP detection was performed using KSNP (v.3.1, *k* = 19 [56–58]) with the assembled isolates (*n* = 2,797) and reference genomes retrieved from NCBI (*n* = 56). The resulting core SNP matrix fasta file was processed in MEGA (v.10.2.6 [27]) to construct a neighbor-joining phylogenetic tree with 50 bootstrap replicates. The tree was further visualized and annotated in iTOL (30, 59). This phylogenetic tree, isolate assembly statistics (percent G + C [51.9%–52.5%], total length [4.5–5.0 Mb], number of contigs [22–217], and estimated coverage [20–275×]), and previous studies (54) were used to develop the inclusion criteria: 51.9%–52.4% G + C, 4.4–5.0 Mb length, ≤200 contigs, and estimated coverage ≥10×. Exclusions (*n* = 10) were made accordingly (15). The remaining isolates (*n* = 2,787) were divided into clades.

Most of the *S*. Mississippi clinical isolates were within clade Ai (*n* = 2,761). KSNP was rerun with only clade Ai isolates for increased resolution of genomic distance between the clinical isolates. The resulting core SNP matrix fasta file was used to create a phylogenetic tree as previously described. FastBAPS (v.1.0.8, initial hierarchy algorithm [64]) was used to systematically determine subclades within clade Ai, and KSNP was run separately with each of the seven resulting clade Ai subclades (Ai1–Ai7). The resulting core SNP matrix fasta files were used to create phylogenetic trees as previously described.

### Mapping county-level IR

County-level IR of *S*. Mississippi (*n* = 2,667) was mapped using ArcGIS Pro (v.3.3 [65]) and TIGER/Line shapefiles containing population data for the year 2020 from the United States Census Bureau FTP Archive (66). Census data from 2020 was selected because 2020 is the midpoint between 2017 and 2022, during which most of the isolates were collected (33). A total of 72 isolates could not be included in the spatial analysis due to missing source county. The metadata of these isolates do not have the geographical linkage required to perform a spatial analysis. County-level IR was computed by dividing the total number of sequenced isolates from each county by the county population and applying a population multiplier of 1,000. The county-level spatial distribution of the isolates within clade Ai was mapped. Individual maps for each subclade were generated, and their spatial patterns were visually compared.

### Measuring county-level spatial clustering

Esri shapefiles for clade Ai and all subclades (Ai1–Ai7) were imported into GeoDa (v.1.20.0.10) (67). Five-digit FIPS codes were used to create a first-order queen contiguity spatial weights matrix. The spatial weights and global Moran’s *I* (68) were used to test for county-level spatial autocorrelation (69) of *S*. Mississippi isolates, observing both the univariate and EB adjusted test statistics for clade Ai and each subclade. For the EB adjusted calculation, county population was used as the base variable. Significance testing was performed using 99 Monte Carlo iterations with significance assessed at *P* < 0.05.

### Univariate analysis

Data processing and statistical analysis were conducted using R (v.4.3.2 [70]) in RStudio (v.2023.12.1+402 [71]). The tidyverse library (72) was used to clean, parse, and analyze data for geographical and genomic distance for clade Ai. Geographical distance was defined as the pairwise distance between the geographical centroids of counties of isolation for isolates in the study population. All combinations of pairwise county-to-county distances were retrieved from the National Bureau of Economic Research. These distances are great-circle distances, calculated using the Haversine formula (73). Pairwise distances for county and core SNP data were cleaned, with geographical data restricted to counties within the states of AL, AR, GA, KY, LA, MS, NC, SC, TN, and VA for isolates with a known county of origin. Isolates with no known county of origin were excluded. County distances were converted from miles to kilometers. Genomic distance was defined as the pairwise core SNP difference between individual isolates; pairwise core SNP differences were calculated using MEGA and exported in CSV format. Isolate identifiers (SRR IDs) were parsed together with their respective county of isolation (five-digit FIPS code) to combine county and core SNP difference data. The psych library [psych::describe() (74)] was used to better understand the variables’ distributions. Diagnostic plots were retrieved using lm() to review diagnostic plots. The residuals and variance of the regression model were not normally distributed, violating the assumptions of normality and homoscedasticity. To ensure the assumptions of SLR are met, we used the Box-Cox transformation method to systematically identify the appropriate power transformation for the dependent variable, geographical distance (km). The MASS library [MASS::bc()] (75) was used to perform Box-Cox analysis (76, 77). The Box-Cox method suggested the square root transformation on the response variable, geographical distance (km) for clade Ai and subclades Ai1, Ai2, Ai3, Ai5, Ai6, and Ai7, and suggested the log transformation for subclade Ai4 (Table 3). The regression coefficients following transformation represent the change in the square root of the dependent variable per unit change in the predictor variable.

### Regression analysis

SLR was performed to provide an assessment of association. Scatterplots were generated using plot() + abline() to visually assess for correlation, with genetic distance (no. core SNPs) on the *x*-axis and geographical distance (km) on the *y*-axis (Fig. S2). The scatterplot for subclade Ai4 is shown separately because there were only six observations (Fig. S1). Correlation analysis was performed using cor.test() to assess the correlation between the outcome and explanatory variables. After transforming geographical distance based on the lambda provided by bc() (Table 3) and reassessing the distribution with hist(), lm() was used to perform SLR to measure the association between the square root of pairwise geographical distance in kilometers (response/dependent variable) and pairwise genomic distance in SNP differences (predictor/independent variable) for the *S*. Mississippi clinical isolates. This process was repeated on individual data frames using core SNP differences from individual KSNP runs for each of the seven subclades. A second data frame for clade Ai was constructed by parsing together individual subclade data frames using rbind(). Unlike the initial data frame for clade Ai, this data frame contained only within-group pairs. Bonferroni correction was applied by adjusting the model *P* value for each of the seven subclades (*P* × *m*, where *m* = 7).

## SUPPLEMENTAL MATERIAL

Figure S1 aem.02136-25-s0001.pdf County-level incidence of subclade Ai4 *S*. Mississippi clinical isolates and scatterplot of genomic distance vs geographical distance between isolates.

Figure S2 aem.02136-25-s0002.pdf Scatterplots of genomic distance vs geographical distance between *S*. Mississippi isolates.

Figure S3 aem.02136-25-s0003.pdf Flowchart showing method details and isolate numbers for phylogenetic analyses.

Tables S1 and S2 aem.02136-25-s0004.xlsx Table S1, study isolate details; Table S2, reference isolate details.

ASM does not own the copyrights to Supplemental Material that may be linked to, or accessed through, an article. The authors have granted ASM a non-exclusive, world-wide license to publish the Supplemental Material files. Please contact the corresponding author directly for reuse.
