# Supplementary material for: Phylogeographic clustering of Salmonella enterica serovar Mississippi in the southeastern United States indicates regional transmission pathways
Source: Appl Environ Microbiol. 2026 Jan 27;92(2):e02136-25. doi: 10.1128/aem.02136-25 (PMC12915305; doi:10.1128/aem.02136-25)
Supplement: Figure S1 — County-level incidence of subclade Ai4 S. Mississippi clinical isolates and scatterplot of genomic distance vs geographical distance between isolates. [file aem.02136-25-s0001.pdf]

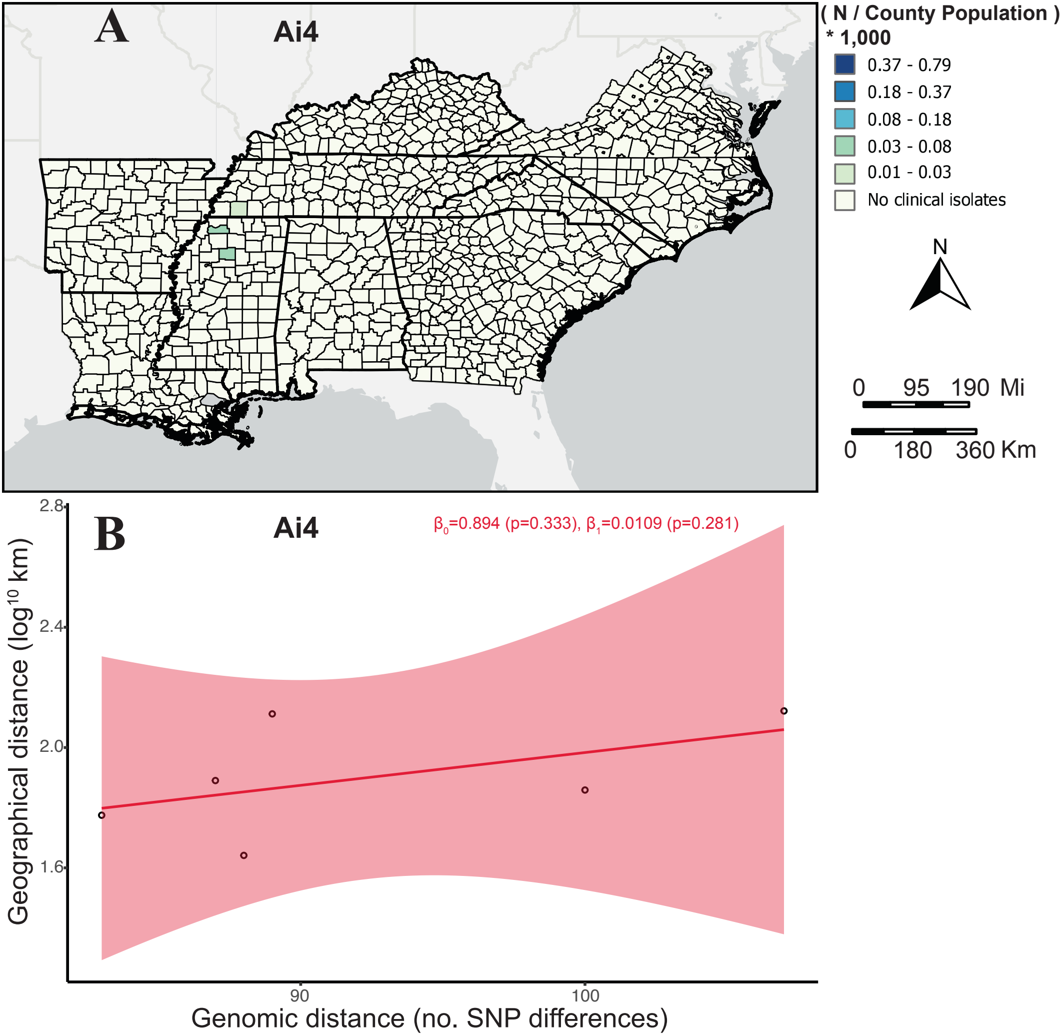

Figure S1. County-level incidence of subclade Ai4 *S.* Mississippi clinical isolates and scatterplot of genomic distance vs. geographic distance between isolates.

(**A**) Counties are shaded by number of *S.* Mississippi isolates per county population (see legend at top right) for subclade Ai4. This includes all relevant study isolates. (**B**) Scatterplot showing genomic distance (no. core SNP differences) vs. geographic distance (km, log transformed) for subclade Ai4. The red line and light red shading represent the regression line and 99% confidence interval of the simple linear model. The y-intercept (β_0), slope (β_1), and their associated p-values are in red at the top right. F statistic and model p-value are provided in **Table 3**.
