# Supplementary material for: Phylogeographic clustering of Salmonella enterica serovar Mississippi in the southeastern United States indicates regional transmission pathways
Source: Appl Environ Microbiol. 2026 Jan 27;92(2):e02136-25. doi: 10.1128/aem.02136-25 (PMC12915305; doi:10.1128/aem.02136-25)
Supplement: Figure S2 — Scatterplots of genomic distance vs geographical distance between S. Mississippi isolates. [file aem.02136-25-s0002.pdf]

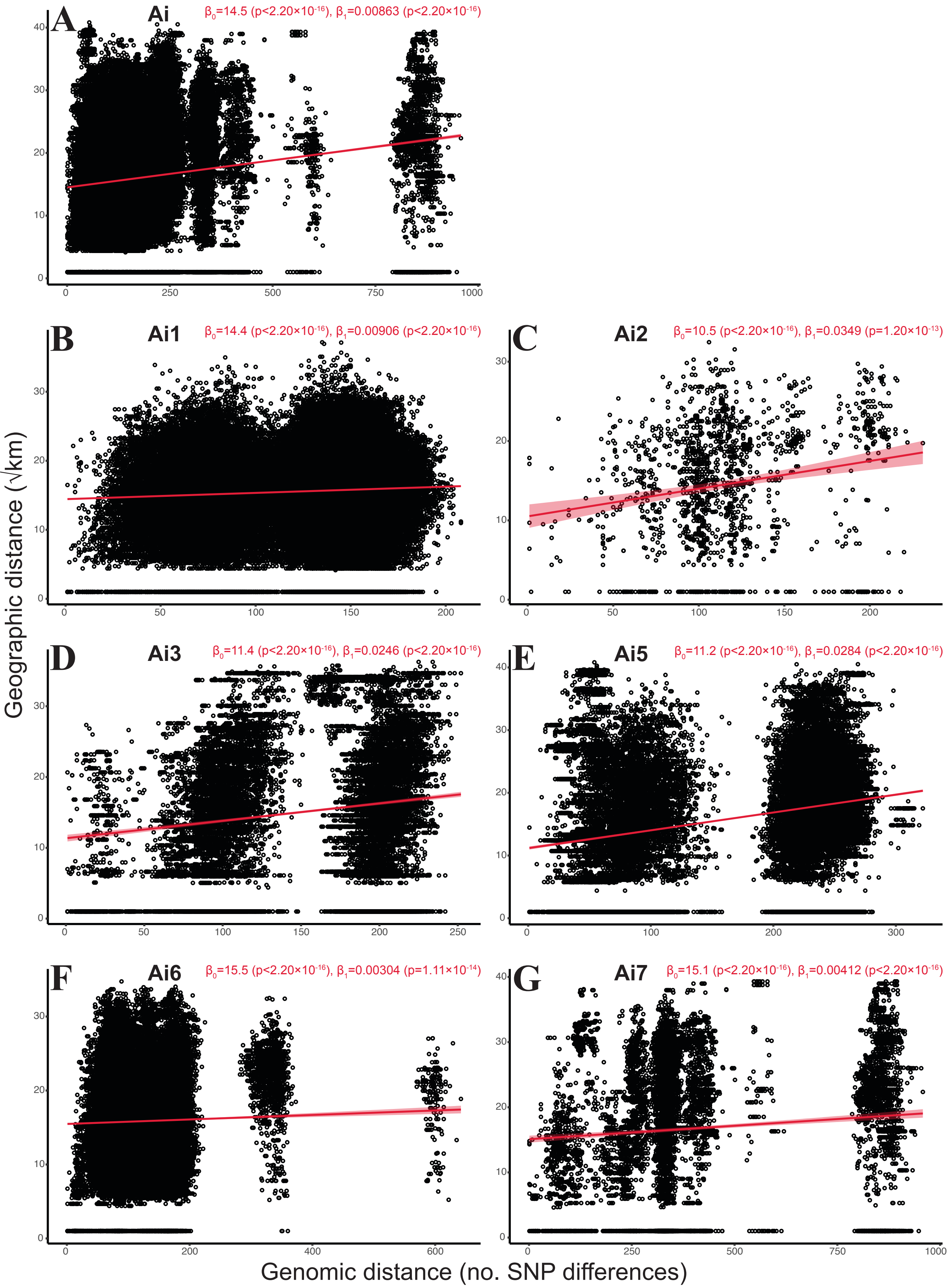

Figure S2. Scatterplots of genomic distance vs. geographic distance between *S.* Mississippi isolates.

Scatterplots showing genomic distance (no. core SNP differences) vs. geographic distance (km, square root transformed) for (**A**) all of clade Ai and (**B-G**) subclades Ai1 through Ai7, excluding subclade Ai4. For each, the red line and light red shading represent the regression line and 99% confidence intervals of the simple linear model. The y-intercept (β_0), slope (β_1), and their associated p-values are in red at the top right of each panel. F statistics and model p-values are provided in **Table 3**.
