# Supplementary material for: Phylogeographic clustering of Salmonella enterica serovar Mississippi in the southeastern United States indicates regional transmission pathways
Source: Appl Environ Microbiol. 2026 Jan 27;92(2):e02136-25. doi: 10.1128/aem.02136-25 (PMC12915305; doi:10.1128/aem.02136-25)
Supplement: Figure S3 — Flowchart showing method details and isolate numbers for phylogenetic analyses. [file aem.02136-25-s0003.pdf]

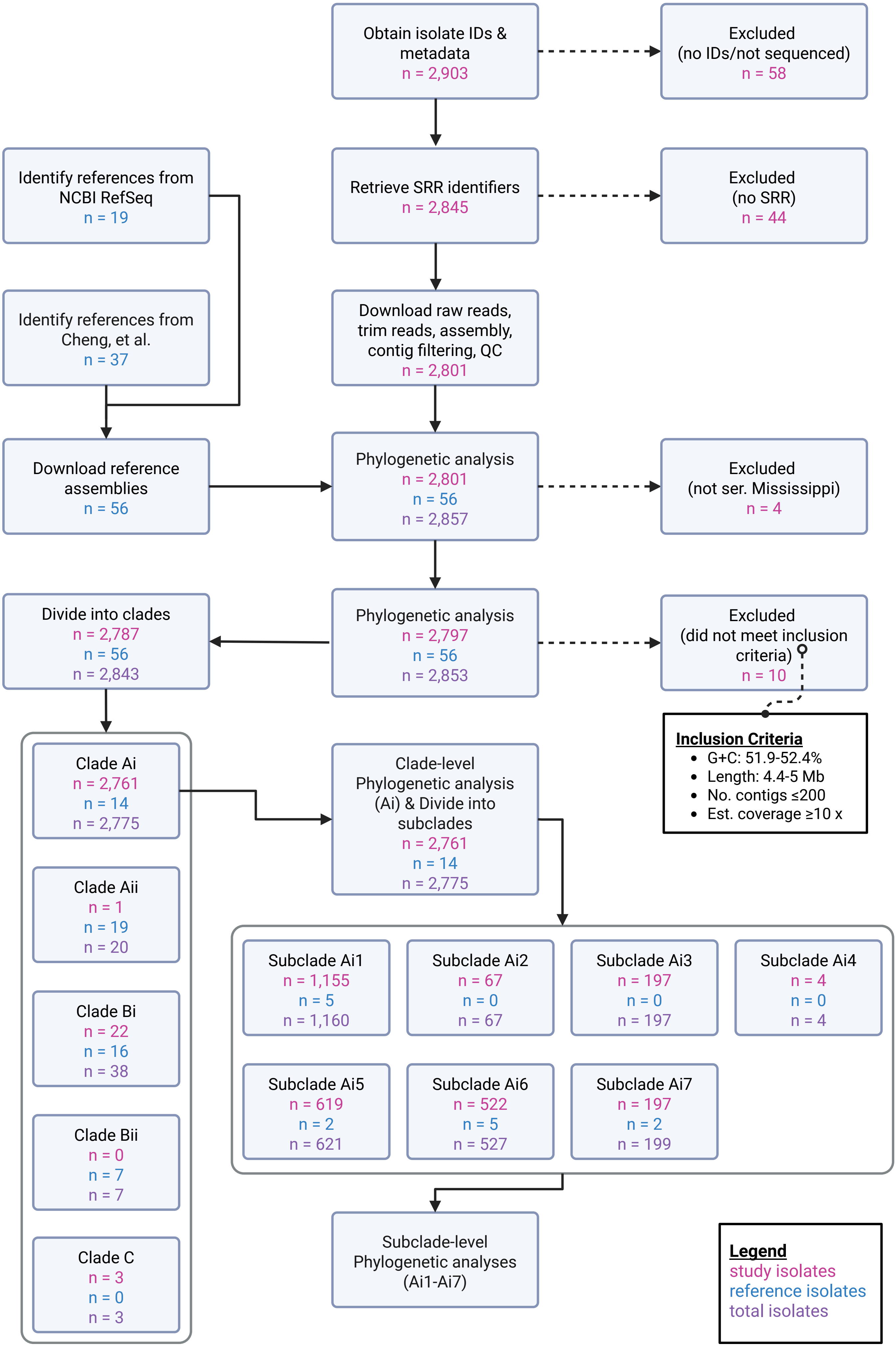

Figure S3. Flowchart showing method details and isolate numbers for phylogenetic analyses. Flowchart created in BioRender(https://BioRender.com)
